# A first probable case of SARS-CoV-2 reinfection in Colombia

**DOI:** 10.1186/s12941-020-00413-8

**Published:** 2021-01-12

**Authors:** Whilken Novoa, Hollman Miller, Salim Mattar, Álvaro A. Faccini-Martínez, Ricardo Rivero, Hector Serrano-Coll

**Affiliations:** 1 Secretaria de Salud del Vaupes, Mitu, Colombia; 2 Universidad de Cordoba, Instituto de Investigaciones Biologicas del Tropico, Monteria, Colombia

Although reinfection by SARS-CoV-2 is a rare phenomenon, cases with suspected or possible reinfection by SARS-CoV-2 have recently been reported in different countries. In some cases, it is not clear whether the individual's RT-qPCR test remained positive for a prolonged period after the first episode of infection or whether it represents true reinfection. The duration of viral RNA is variable in samples from the upper respiratory tract; RNA has been detected up to 104 days after the onset of symptoms [1]. To date, 8 cases of reinfection have been reported, 2 in India, one in Hong Kong, Belgium, Nevada, Ecuador, and the Netherlands. It is not clear why reinfections occur, but it could be due to the decrease in antibody levels, which are more decreased in asymptomatic patients, although in the eight patients reported so far, 4 had symptoms, and one was hospitalized [1].

This study describes the first probable case of SARS-CoV-2 reinfection in Colombia. On June 26, 2020, a 44-year-old male patient, immunocompetent, without comorbidities, a health care worker had contact with a symptomatic relative with a positive RT-qPCR test (Fig. 1). On July 2, the patient and his family underwent RT-qPCR testing from nasopharyngeal specimens, giving a positive result for SARS-CoV-2 (Cq 36.89). He and his family remained asymptomatic. Then, on July 18, a new RT-qPCR test was performed, and the result was negative (Fig. 1). On September 17, the patient underwent an antigen test required for a domestic flight in Colombia; the result was negative. On September 20, the patient moved from the first place of infection 500 km away to another municipality bordering Brazil. He is a health care worker involved in public health activities related to the COVID-19 pandemic. On October 11, the patient-reported symptoms such as malaise, chills, headache, fever, and odynophagia. Afterward, on October 15, oropharyngeal and nasopharyngeal samples were taken for antigen detection and RT-qPCR testing, respectively; both tests were positive. No samples were taken from the patient to other respiratory viruses complex because they are not performed routinely in patients with COVID-19. On October 26, the patient continued with symptoms and recovered at home (Fig. 1).

**Fig. 1:**
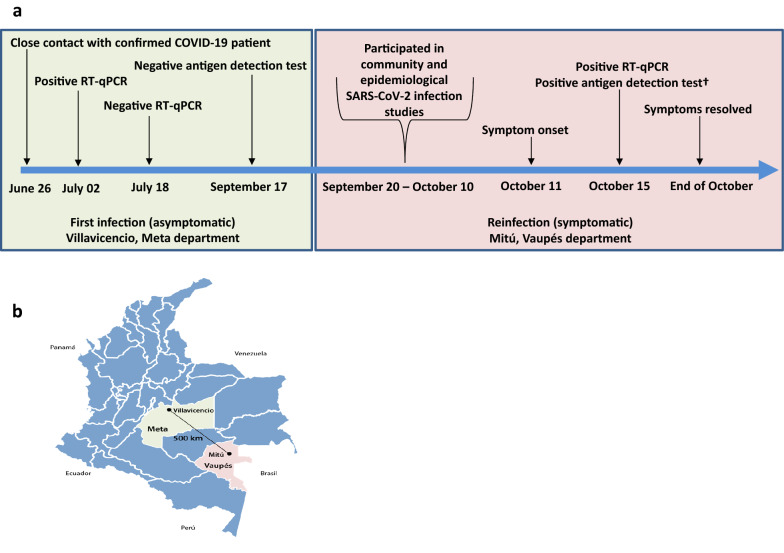
**a** Case timeline. The patient had close contact with a confirmed COVID-19 patient on June 26, then tested positive for SARS-CoV-2 by RT-qPCR on July 02 (asymptomatic). He was followed by two negative diagnoses (RT-qPCR and antigen tests) on July 18 and September 17. Between September 20 and October 10, the patient participated in community SARS-CoV-2 infection studies, having symptom onset on October 11 (malaise, odynophagia, rhinorrhea, cough, dyspnea, and fever 38.7 °C). On October 15, the patient tested positive for RT-qPCR having symptom resolution by October 25. **b** Geographical location of the infection episodes (1st episode: Villavicencio, Meta; 2nd episode: Mitú, Vaupés, places are distanced by 500 km)

Although next-generation sequencing was not carried out to establish the phylogenetic patterns of the two strains in this report, the case shows that reinfection is possible in Colombia and that probably the duration of the antibodies is shorter than expected [2]. That was shown by Liu et al. [3], who showed that specific antibodies against SARS CoV2 disappeared three months after the symptoms' onset. Long et al. [4] also evidenced that in asymptomatic antibodies neutralizing decline in 40% of the asymptomatic individuals. Furthermore, epidemiological links show that reinfection could be occurring in different parts of Colombia, where the press has reported anecdotal cases. Although the present case does not have a genetic analysis of the virus, it suggests a strong epidemiological and microbiological result. The obtaining of two RT-qPCR test positive results at different times, cities and laboratories, shows that we may be facing a case of reinfection in Colombia.

Furthermore, a negative sample was obtained a month before the second RT-qPCR, and the case is linked to a health care worker who is at daily risk. Unfortunately, we do not have the Cq value of the second RT-qPCR, which is a limitation of our work. On the other hand, transient immunity represents a warning for those health care workers who have been previously infected. However, it is relevant to mention that the risk of reinfection in this population would be low, given that the rate of infection by SARS-CoV2 in health workers is around 10% [5, 6]. Whereas, serology is not carried out in Colombia on a routine basis because the country has more than 1,270,991 cases, and its health system is resisting with limited resources the push of this first peek of the pandemic. Currently, the fatality rate reaches 2.81% (35.680/1.270.991), and although there is a decrease in cases in some departments, the increase in Colombia's number of cases is significant in epidemiological terms.

On the other hand, we have a second hypothesis that the patient was reinfected by another strain of SARS-CoV2, given that his new infection occurred in a municipality near Brazil, although we cannot assure that it is a genetically different virus. A study conducted in the northern area of Brazil found a patient infected with a virus variant from lineage A. This variant had a total of 9 mutations in comparison to the reference genome (NC_045512.2), being 4 of them non-silent mutations [7, 8]. Nonetheless, phylogenetic analysis in Colombia showed that patients from the Meta department (location of the first episode) were infected with a virus belonging to lineage B1 [6]. Mutant viruses due to variations in spike protein have been found to evade neutralizing antibodies [9].

Finally, previous exposure to SARS-CoV-2 may not guarantee full immunity in all cases. People either previously diagnosed with COVID-19 or not should be taking the same precautions to avoid SARS-CoV-2 infection. The implications of reinfections are a currently important topic in phase III of the research in vaccines' application.

## Data Availability

No apply.
